# Supplementary material for: Tibiofemoral contact force differences between flat flexible and stable supportive walking shoes in people with varus-malaligned medial knee osteoarthritis: A randomized cross-over study
Source: PLoS One. 2022 Jun 2;17(6):e0269331. doi: 10.1371/journal.pone.0269331 (PMC9162314; doi:10.1371/journal.pone.0269331)
Supplement: S1 Table — Data reported as (Mean (SD) of groups and mean (95% CI) change within and between groups, adjusted for baseline scores. (DOCX) [file pone.0269331.s002.docx]

| **Supplementary Table 1.** Root mean square error (Nm·kg) for CEINMS predicted knee flexion/extension moments compared to OpenSim generated inverse dynamics. Data reported as (Mean (SD) of groups and mean (95% CI) change within and between groups, adjusted for baseline scores. | | | | | | | | |
| --- | --- | --- | --- | --- | --- | --- | --- | --- |
|  |  | Flat flexible  (n = 28) | Stable supportive (n=28) |  | Mean difference (95%CI)  Stable supportive minus Flat flexible | |  |  |
| Stance |  | 0.20 (0.04) | 0.20 (0.04) |  | 0.00 (-0.01, 0.00) |  | |  |
| Swing |  | 0.08 (0.02) | 0.08 (0.02) |  | 0.00 (0.00, 0.01) |  | |  |
| Cycle |  | 0.16 (0.03) | 0.16 (0.03) |  | 0.00 (-0.01, 0.00) |  | |  |
|  | | | | | | | | |
