# Supplementary material for: Tibiofemoral contact force differences between flat flexible and stable supportive walking shoes in people with varus-malaligned medial knee osteoarthritis: A randomized cross-over study
Source: PLoS One. 2022 Jun 2;17(6):e0269331. doi: 10.1371/journal.pone.0269331 (PMC9162314; doi:10.1371/journal.pone.0269331)
Supplement: Attachment — Submitted filename: Response to Reviewers.docx [file pone.0269331.s008.docx]

We thank the reviewers for feedback and suggestions. Please see below for our responses for each of the reviewers’ comments including accompanying amendments to the manuscript. We have combined comments of similar nature where possible to reduce repetition.

**Editor**

**Editor:** Please ensure that your manuscript meets PLOS ONE's style requirements, including those for file naming. The PLOS ONE style templates can be found at

https://journals.plos.org/plosone/s/file?id=wjVg/PLOSOne_formatting_sample_main_body.pdf and

https://journals.plos.org/plosone/s/file?id=ba62/PLOSOne_formatting_sample_title_authors_affiliations.pdf”

**Response:** Thank you for providing these templates. We have modified our manuscript and supporting documents to be consistent with PLOS ONE’s style requirements.

**Amendment:**

Title page: removed running title, reformatted author affiliations

Files have been renamed accordingly

Changes to text formatting: first line indentation, font sizes of headings.

Added supporting information section at end of manuscript containing supplementary data titles and legends.

**Editor:** Thank you for stating the following in the Funding Section of your manuscript:

“This project was supported by funding from the Australian National Health and Medical Research Council (NHMRC) (Project Grant #1124418). SCS is supported by an Australian Government Research Training Program Scholarship. MH is supported by an NHMRC Investigator Grant (#1172928). RSH is supported by a NHMRC Senior Research Fellowship (#1154217). KLP is supported by NHMRC Emerging Leadership Investigator Grant (#1174229). The study sponsors did not play any role in the study design, data collection, analysis and interpretation of the data, writing of the manuscript or the decision to submit the manuscript for publication.” We note that you have provided funding information that is not currently declared in your Funding Statement. However, funding information should not appear in the Acknowledgments section or other areas of your manuscript. We will only publish funding information present in the Funding Statement section of the online submission form. Please remove any funding-related text from the manuscript and let us know how you would like to update your Funding Statement. Currently, your Funding Statement reads as follows: “This project was supported by funding from the Australian National Health and Medical Research Council (NHMRC) (Project Grant #1124418). SCS is supported by an Australian Government Research Training Program Scholarship. MH is supported by an NHMRC Investigator Grant (#1172928). RSH is supported by a NHMRC Senior Research Fellowship (#1154217). KLP is supported by NHMRC Emerging Leadership Investigator Grant (#1174229). The study sponsors did not play any role in the study design, data collection, analysis and interpretation of the data, writing of the manuscript or the decision to submit the manuscript for publication.” Please include your amended statements within your cover letter; we will change the online submission form on your behalf.

**Response:** Thank you for identifying this oversight. We have removed the funding statement from the manuscript and have added it to the cover letter. We do not request any amendments to the funding statement given it is consistent with that now provided in the cover letter.

**Amendment**:

Cover letter - “This project was supported by funding from the Australian National Health and Medical Research Council (NHMRC) (Project Grant #1124418). SCS is supported by an Australian Government Research Training Program Scholarship. MH is supported by an NHMRC Investigator Grant (#1172928). RSH is supported by a NHMRC Senior Research Fellowship (#1154217). KLP is supported by NHMRC Emerging Leadership Investigator Grant (#1174229). The study sponsors did not play any role in the study design, data collection, analysis and interpretation of the data, writing of the manuscript or the decision to submit the manuscript for publication.”

**Editor:** In your Data Availability statement, you have not specified where the minimal data set underlying the results described in your manuscript can be found. PLOS defines a study's minimal data set as the underlying data used to reach the conclusions drawn in the manuscript and any additional data required to replicate the reported study findings in their entirety. All PLOS journals require that the minimal data set be made fully available. For more information about our data policy, please see http://journals.plos.org/plosone/s/data-availability.

Upon re-submitting your revised manuscript, please upload your study’s minimal underlying data set as either Supporting Information files or to a stable, public repository and include the relevant URLs, DOIs, or accession numbers within your revised cover letter. For a list of acceptable repositories, please see http://journals.plos.org/plosone/s/data-availability#loc-recommended-repositories. Any potentially identifying patient information must be fully anonymized.

Important: If there are ethical or legal restrictions to sharing your data publicly, please explain these restrictions in detail. Please see our guidelines for more information on what we consider unacceptable restrictions to publicly sharing data: http://journals.plos.org/plosone/s/data-availability#loc-unacceptable-data-access-restrictions. Note that it is not acceptable for the authors to be the sole named individuals responsible for ensuring data access.

We will update your Data Availability statement to reflect the information you provide in your cover letter.

We note that you have indicated that data from this study are available upon request. PLOS only allows data to be available upon request if there are legal or ethical restrictions on sharing data publicly. For information on unacceptable data access restrictions, please see http://journals.plos.org/plosone/s/data-availability#loc-unacceptable-data-access-restrictions.

In your revised cover letter, please address the following prompts:

a) If there are ethical or legal restrictions on sharing a de-identified data set, please explain them in detail (e.g., data contain potentially identifying or sensitive patient information) and who has imposed them (e.g., an ethics committee). Please also provide contact information for a data access committee, ethics committee, or other institutional body to which data requests may be sent.

b) If there are no restrictions, please upload the minimal anonymized data set necessary to replicate your study findings as either Supporting Information files or to a stable, public repository and provide us with the relevant URLs, DOIs, or accession numbers. Please see http://www.bmj.com/content/340/bmj.c181.long for guidelines on how to de-identify and prepare clinical data for publication. For a list of acceptable repositories, please see http://journals.plos.org/plosone/s/data-availability#loc-recommended-repositories.

We will update your Data Availability statement on your behalf to reflect the information you provide.

**Response:**

We have contacted our institutional ethics committee who have now permitted us to share the minimum coded dataset. We have made the discrete and continuous medial tibiofemoral joint contact force datasets available as Supplementary files.

**Amendment**:

S1 File: Stance phase medial tibiofemoral contact force for flat flexible and stable supportive shoes (CSV)

S2 File: Stance phase muscle contribution to medial tibiofemoral contact force for flat flexible and stable supportive shoes (CSV)

S3 File: Stance phase external contribution to medial tibiofemoral contact force for flat flexible and stable supportive shoes (CSV)

S4 File: Walking speed, peak medial tibiofemoral joint contact force, loading and stance phase medial joint contact impulse and loading rate (mean and max) for flat flexible and stable supportive shoes (CSV)

**Reviewer #1**

**Reviewer #1:** A randomized clinical trial with a crossover study design aimed to compare the effect of stable supportive to flat flexible walking shoes on medial tibiofemoral joint contact force (MTCF) in individuals with medial knee osteoarthritis and varus malalignment. Waveforms were analyzed using parametric mapping with repeated measures ANOVA. Discrete outcomes were compared using repeated measures multivariate ANOVA models. Statistical parametric mapping showed a statistically significant difference between the two conditions. Loading impulse was significantly lower in supportive shoes compared to flexible ones.

**Response:** Thank you for your overall positive feedback.

**Reviewer #1:** Abstract: Improve the clarity of the objective statement. "To compare the effect of stable supportive shoes to flat flexible walking shoes...".

**Response:** We have now implemented this suggestion

**Amendment**:

Abstract – Line 3: “To compare the effect of stable supportive to flat flexible walking shoes on medial tibiofemoral contact force (MTCF) in people with medial knee osteoarthritis and varus malalignment.”

**Reviewer #1:** Table 1: The notation for mean +/- SD has not been utilized to display the values in the table.

**Response:** Thank you for highlighting this oversight. For clarity, we have now modified this sentence by removing the abbreviation.

**Amendment**:

Table 1: “Except where indicated otherwise, values are mean (standard deviation)”

**Reviewer #1:** The standard statistical term for average is mean.

**Response:** We have now changed all instances of “average” to “mean”, predominantly relating to the outcome “average loading rate”.

**Amendment**:

Changed “average” to “mean” on lines 23, 256, 258, 281, 286, 288, 315, 331, 342, 361, 371, 381, 387, 394, 490, Table 1, Table 2 and Table 3.

**Reviewer #2**

**Reviewer #2:** Thank you for the opportunity to review this manuscript. The manuscript " The immediate effect of flat flexible vs stable supportive walking shoes on tibiofemoral contact force in people with varus-malaligned medial knee osteoarthritis (PONE-D-21-29346)" contains interesting data that allow to understand the possible advantages and disadvantages of using different types of shoes in people with varus-malaligned medial knee osteoarthritis, from a complex biomechanical point of view and with with an important innovation using a novel insight from our use of neuromusculoskeletal modelling is the evaluation of the absolute muscular and external load components of MTCF.

I think these data would make a nice contribution to our knowledge on the type of shoes recommendation, based on biomechanics responses and would fit into PlosOne. The experimental approach and the methods of the study seem mostly sound, especially the idea about the integrative view of different biomechanical signals: kinematic, kinetic and electromyographic. However, in the current state I consider the manuscript partly incomprehensive. I have several rather general suggestions because I think the manuscript requires several important changes to improve the overall scientific question.

**Response:** Thank you for your review, with great attention to detail. We appreciate your acknowledgement of the complexity of our neuromusculoskeletal modelling.

**Reviewer #2:** Title: I would like to suggest a change to the title, in order to make it more understandable: “Tiobiofemoral contact force differences between flat flexible and stable supportive walking shoes in people with varus-malaligned medial knee osteoarthritis.”

**Response:** We have now implemented this suggestion, with the addition of “a randomised cross-over study” at the end of the title to provide clarity on the study design.

**Amendment**:

Title page: “Tibiofemoral contact force differences between flat flexible and stable supportive walking shoes in people with varus-malaligned medial knee osteoarthritis: a randomized cross-over study.”

**Reviewer #2:** Running title suggestion: Comparing tibiofemoral loads with different types of shoes in varus-malaligned osteoarthritis.

**Response:** Thank you for this suggestion. We have now removed the running title from the manuscript, given the PLOS ONE title page formatting guidelines does not request one be provided.

**Amendment**:

Running title has been removed from the title page of the manuscript.

**Reviewer #2:** Review: “Lower body motion”. In methods, full-body appears. There is important confusion in the description of the kinematic method used. It is necessary to explain in detail which reference was used for the construction of the kinematic model, since sixty-seven reflective markers were used, and the presented reference (number 27) uses a simpler model, only with lower-limb markers: “.. .using each thigh, shank, and foot segment cluster...".

**Response:** Thank you for highlighting this ambiguity. Indeed, a full-body set of 67 marker trajectories were obtained during data analysis (including clusters on the shank, thigh, forearm and upper arm). However, only the lower-limb and pelvis markers were used to conduct the biomechanical analysis as per Besier et al. 2003 *J Biomech* <https://doi.org/10.1016/S0021-9290(03)00087-3>). Only markers on the lower-limb and pelvis are necessary for the standard inverse kinematics/dynamics approach used to calculate ankle, knee and hip angles and moments in this study. The upper limb marker trajectories are superfluous for the purpose of this manuscript and were collected to investigate trunk and upper limb compensatory strategies in complementary studies. For clarity, we have changed lower-body to full-body motion in the abstract.

**Amendment**:

Abstract – Line 10: “Three-dimensional full-body motion, ground reaction forces and surface electromyograms from twelve lower-limb muscles were acquired”

**Reviewer #2:** Suggestion to change the text of the results: from “Statistical parametric mapping showed a significant effect between the two test conditions (p=0.001), with post-hoc tests showing lower MTCF in stable supportive compared to flat flexible shoes during 5-18% of stance phase.”; to “Statistical parametric mapping showed lower MTCF in stable supportive compared to flat flexible shoes during 5-18% of stance phase.”

**Response:** We have now implemented this suggestion, with the addition of the p value for statistical clarity.

**Amendment**:

Abstract – Line 20-21: “Statistical parametric mapping showed lower MTCF in stable supportive compared to flat flexible shoes during 5-18% of stance phase (p=0.001).”

**Reviewer #2:** Insert information regarding incidence rates of medial knee osteoarthritis to further justify the clinical relevance of the specific choice of this research sample.

**Response:** We have implemented as suggested.

**Amendment**:

Introduction – Line 40-44: “Knee osteoarthritis (OA), predominantly of the medial tibiofemoral compartment (1), is a highly prevalent and disabling global chronic disease affecting 1 in 5 people over the age of 40 (2). Pathogenesis of medial knee OA is thought to be due in part to abnormal joint loading within the medial tibiofemoral compartment, with estimates of medial tibiofemoral contact force (MTCF) having been associated with structural disease progression in knee OA (3).”

**Reviewer #2:** Insert a sentence at the end of the first paragraph that serves as a link to develop the subject about the type of shoe.

**Response:** We have implemented as suggested.

**Amendment**:

Introduction – Line 49-51: “As there is no cure for OA, appropriate self-management strategies are strongly advocated to reduce symptoms and delay the need for arthroplasty (9, 10). Footwear is a low-burden simple self-management strategy.”

**Reviewer #2:** Start a new paragraph after the end of line 49.

**Response:** We have now implemented this suggestion.

**Reviewer #2:** Suggestion for changing the goal text: from “The aim of this study was to compare the effects of flat flexible and stable supportive walking shoes on MTCF waveforms using and discrete measures of MTCF and MTCF loading rate in people with medial knee OA and varus malalignment.”; to “The aim of this study was to compare the effects of flat flexible and stable supportive walking shoes on MTCF waveforms, discrete measures of MTCF and MTCF loading rate in people with medial knee OA and varus malalignment.”

**Response:** The authors agree that refinement of aim statement was necessary. As loading rate is considered a discrete measure of MTCF, we have modified the reviewer’s suggestion slightly to reflect this, rather than report loading rate as a separate entity.

**Amendment**:

Introduction – Line 89-92: “The aim of this study was to use EMG-informed neuromusculoskeletal modelling to compare the effects of flat flexible and stable supportive walking shoes on MTCF waveforms and discrete measures of MTCF (peak, impulse and loading rate) in people with medial knee OA and varus malalignment.”

**Reviewer #2:** Rewrite the sentence (line 36 – 41), it is too long. Split into two sentences or increase the brevity.

**Response:** We have separated this sentence into two as suggested.

**Amendment**:

Introduction – Line 44-48: “As estimates of tibiofemoral loading are sensitive to frontal plane knee kinematics (4), an important subgroup for consideration is people with medial knee OA and varus malalignment. This subgroup has demonstrated greater functional (5) and structural decline (6) than those with neutrally aligned knees…”

**Reviewer #2:** Does the word “surrogate” (line 52) have any other synonyms? What does it really mean? As it is written, it seems to me an alternative measure to some “gold standard” measure. If so, what would this “gold standard” measure be?

Here I take this opportunity to apologize for any failure in English, as it is not my native language.

**Response:** The reviewer is correct in the interpretation of this term. In this case, the “gold standard” measure is internal tibiofemoral contact force, which is only feasibly measured by instrumented knee prosthetics. This is impossible to do in our population who are pre-operative. In essence, all measures of loading in people with knee osteoarthritis are surrogate measures, which further necessitates the need for accurate, subject specific modelling to best approximate internal contact force.

A synonym also used in the literature is “proxy”. We are happy to substitute at the discretion of the reviewer/editor.

**Amendment**: No amendment

**Reviewer #2:** I suggest rewriting the subtopics in order to separate and specify the “step-by-step” of data acquisition and the “step-by-step” of data processing. The text is a little confusing about this.

**Response:** We have now separated the data acquisition and data processing methods into respective headings and sub-headings.

**Amendment**:

Addition of headings/subheadings within methods:

Methods – Line 152: “Data acquisition”

Methods – Line 192: “Data pre-processing”

Methods – Line 204: “Fig 2: Flowchart outlining the data processing methods.”

Methods – Line 206: “Data processing”

Methods – Line 208: “Musculoskeletal modelling”

Methods – Line 226: “Calibrated-informed neuromusculoskeletal modelling”

Methods – Line 249: “Outcomes”

**Reviewer #2:** Check spelling of the word “familiarisation” (line 134)

**Response:** We have now modified all words using British English to American English, including “familiarization”.

**Amendment**:

Line 8,97: “randomised” to “randomized”

Line 163,181: “standardised” to “standardized”

Line 14, 133, 135: “analysed” to “analyzed”

Line 150: “familiarisation” to “familiarization”

**Reviewer #2:** What is the analysis area size, especially the distance traveled between two photoelectric beams? Was there any space for initial acceleration and final deceleration outside the collection area between the two photoelectric beams?

**Response:** We have now clarified that we used a 10m walkway with photo-electric beams placed mid-way along walkway providing participants with ample space outside the collection area.

**Amendment**:

Methods – Line 158-160: “Participants walked along a 10m walkway at a self-selected speed matched to ±5% across conditions, measured using two photoelectric beams positioned midway along the walkway.”

**Reviewer #2:** It is necessary to describe the processing of kinematic and kinetic data (obtained from the platforms). Was there any use of a standard Pipeline or was there a specific one created based on other references? What was the data filtering strategy? Was there a residual analysis of the signal? What software was used for these processes?

**Response:** The authors agree with the reviewer that further detail is warranted. We used a toolbox in Matlab (MoToNMS - Mantoan et al., 2015 *Source Code Bio Med 10.1186/s13029-015-0044-4*) to convert the motion capture c3d files into trace and motion files for use in OpenSim. We used the standard OpenSim IK, ID and muscle analysis pipelines to determine the lower-limb joint kinematics, moments and muscle-tendon unit kinematics as outlined in Delp et al. (*J Biomech* 2007 10.1109/TBME.2007.901024).

The Delp methods include a least-squares problem that minimises the differences between the measured marker locations and the model’s virtual marker locations, subject to physiological joint constraints.

We have also clarified the use of a low-pass filter at 6Hz cut-off for model kinematics, which are the default parameters recommended by Delp et al. and others (Crenna et al., 2021 *Sensors* doi:10.3390/s21134580).

**Amendment**:

Methods – line 193-195: “A force plate threshold of 10 N was used to define heel-strike and toe-off events, obtained using Vicon Nexus software and checked manually by the researcher.”

Methods – line 195-198: “Laboratory force plate, marker, and EMG data were pre-processed within Matlab 2019b (MathWorks, Massachusetts, USA) using the MOtoNMS toolbox (31). This toolbox processes experimental C3D files from Vicon Nexus and coverts the data into OpenSim file formats (i.e., trace and motion files) (32).”

Methods – line 209-210: “A generic, full-body musculoskeletal model (33) was used within OpenSim (Version 3.3) (32)…”

Methods – line 227-230: “After model scaling, OpenSim inverse kinematics, inverse dynamics, and muscle analysis tools were used to determine the lower-limb joint kinematics, joint moments, and muscle-tendon unit kinematics, respectively (32). Model kinematics were low-pass filtered at a 6Hz cut-off frequency (32, 35).”

**Reviewer #2:** Describe the diameter of the reflective markers.

**Response:** We have clarified that the diameter of the reflective markers were 14mm.

**Amendment**:

Methods – Line 165-167: “A full body marker set, consisting of sixty-seven reflective markers of 14mm diameter were placed on the participant’s skin”.

**Reviewer #2:** How was the Vicon system calibrated? What is the accepted Image error threshold?

**Response:** Thank you for this enquiry. We performed a full calibration at the start of each session using the standardised Vicon 5 reflective marker wand with 10,000 samples across the analysis area (i.e., a 3D rectangle over the force plates). We use a threshold image error of 0.2 for each of the twelve cameras and re-calibrate if the error exceeds this. We set the volume origin using the ErgoCal L-Frame. This was performed as per Vicon specifications. We have now outlined the calibration process in the methods.

We generally do not report this data as the image error varies depending on laboratory environment and the analysis area of interest. Calibration can be performed over a smaller analysis area than that of interest, yielding smaller image errors but causing detrimental effects on the 3D reconstruction.

**Amendment**:

Methods – Line 162-165: “The Vicon system was calibrated using the standardized Vicon 5 reflective marker wand with 10,000 samples across the analysis area. An image threshold error of 0.2 was used for each of the twelve cameras, with recalibration performed if the error exceeded this.”

**Reviewer #2:** I particularly like the EMG data processing logic due to the normalization of the maximum EMG values.

**Response:** We thank the reviewer for acknowledging the robustness of our EMG processing methods, which is considered optimal for this study design.

**Amendment**: No amendment.

**Reviewer #2:** Please, insert the reference to “standardized anatomical landmark coordinates” line 192.

**Response:** To clarify, we measured the bone segments with reference to the landmarks used on the generic model by Rajagopal et al. (2016 *IEE Trans Biomed Eng* 10.1109/TBME.2016.2586891. This standardisation is commonplace as the scale factor is a ratio of the MRI-informed dimensions and the generic model dimensions (Gerus et al., 2013 *J Biomech* 10.1016/j.jbiomech.2013.09.005)

**Amendment**:

Methods – Line 219-222: “Patient-specific scaling of the pelvic, femur and tibia dimensions as well as their mass inertia properties was undertaken by measuring the respective segmented bone lengths and widths using the bony landmarks from the generic model as a reference (33, 34).”

**Reviewer #2:** I like the presentation of the results, however I believe that the information contained in the supplementary tables could be added to the main text, even considering that there is repeated information

**Response:**

We have now moved the results of the KAM variable (S1 Table, S1 Fig and S2 Fig) to the main text. We have modified the outcomes and statistical analysis sections within the methods and added a secondary aim to the introduction accordingly.

S2 Table reports the errors between OpenSim generated inverse dynamics and the CEINMS predicted knee flexion/extension moments. We respectfully disagree that this should be included in the main results section, as it is only used to validate the results of our model.

**Amendment**:

Introduction – Line 92-94: “A secondary aim was to compare the effects of flat flexible and stable supportive walking shoes on KAM waveforms and discrete measures of KAM (peak, impulse and loading rate) in the context of existing research.”

Methods – Line 250-261: “The main load variable of interest was MTCF (including muscle and external components to MTCF), however we also extracted external knee joint moments (i.e., KAM). Load variables over each stance phase of gait were spline interpolated to 101 time points and normalised to bodyweight (BW). The peak (BW) and impulse (BW**^.^**s) were extracted (18, 39). The loading phase of gait was defined as the point from initial contact to the first peak of the load variable (21). The impulse was calculated as time integral of the load variable over the respective time-period. The mean and max loading rate was calculated from existing methods that have evaluated loading rates of the peak vertical GRF (40, 41) and KAM (21, 41). The mean loading rate (BW·s^-1^) was calculated as the load variable peak divided by the time taken from initial contact to the first load variable peak. Within the same time interval, the max loading rate (BW·s^-1^) was determined by calculating the maximum instantaneous slope of the load variable.”

Methods – Line 274-277: “Statistical parametric mapping (SPM) with one-way repeated measures analysis of variance (ANOVA) was conducted within Matlab (43) to examine MTCF waveforms (including muscle and external components) and KAM waveforms for the two shoe conditions (flat flexible and stable supportive).”

Methods – Line 278-279: “If a significant main effect of condition on MTCF or KAM (i.e., suprathreshold cluster existed, p<0.05)…”

Methods – Line 287-289: “Dependent variables in the primary analysis were 1) peak MTCF; 2) MTCF impulse; 3) MTCF loading stance impulse; 4) mean MTCF loading rate; 5) max MTCF loading rate; and 6) walking speed. Dependent variables in the secondary analysis were 1) peak KAM; 2) KAM impulse; 3) KAM loading stance impulse; 4) mean KAM loading rate; 5) max KAM loading rate; and 6) walking speed.”

Results – line 308: “The results of the primary SPM …”

Results – line 325-329: “The results of the secondary SPM with one-way repeated measures ANOVA showed significant suprathreshold clusters between the two shoe conditions for KAM (p<0.001, p=0.008) (Fig 5). Post-hoc testing demonstrated that compared to flat flexible shoes, stable supportive shoes had a lower KAM at 8-17% of stance and a higher MTCF at 26-51% of stance (Fig 6).”

Results – line 331-333: “**Fig 5.** Ensemble mean (± standard deviation) and statistical parametric mapping (using a one-way repeated measures ANOVA) for external knee adduction moment (BW) across the stance phase of gait when walking with stable supportive shoes (green) and flat flexible shoes (yellow)”

Results – line 335-336: “**Fig 6.** Mean differences (95% CI) across shoe conditions based on ANOVA post-hoc comparison of external knee adduction moment (BW).”

Results – line 339: “The results of the primary repeated measures MANOVA …”

Results – line 357-363: “The results of the secondary repeated measures MANOVA showed a significant main effect between shoe conditions (p<0.001). Subsequent post-hoc analysis found that compared to flat flexible shoes, stable supportive shoes had a higher peak KAM (0.04 BW 95%CI [0.02, 0.06], p<0.001) and a higher stance KAM impulse (0.02 BW·s 95%CI [0.01, 0.02], p=0.001) (Table 3). There were no differences observed for KAM loading stance impulse (p=0.36), mean KAM loading rate (p=0.38), max KAM loading rate (p=0.11) or walking speed (p=0.68) (Table 3).”

**Reviewer #2:** The figures that appear in the text file are in low resolution, however the images attached to the links are in good resolution.

**Response:** We speculate that the reviewer has received a compressed manuscript file, given the images within the links are of good resolution. Nonetheless, we have now uploaded all Figures to the PACE digital diagnostic tool, which has modified them to meet the PLOS ONE publishing requirements. We have also checked these figures manually and confirm they have good resolution.

**Reviewer #2:** Please clarify how the data acquisition was carried out in individuals with unilateral OA, considering that 54% of the sample had unilateral symptoms?

**Response:** We have now clarified how participants with unilateral knee OA were analysed.

**Amendment**:

Methods – Line 132-135: “For participants with unilateral symptoms, the unilateral leg was selected as the study knee and data relevant to this limb was analyzed. For participants with bilateral symptoms, the most symptomatic eligible knee was selected as the study knee and data from this limb was analyzed.”

**Reviewer #2:** I suggest a general rewrite in the discussion, because in general it brings a lot of information about your results and speculations than a real discussion with other articles, looking for the mechanisms responsible for the answers found, whether they are in agreement or disagreement, and their reasons.

**Response:**

As this is the first study to evaluate the effects of stable supportive and flat flexible shoes on MTCF during walking using EMG-informed neuromusculoskeletal modelling, direct comparison with existing literature is precluded. All existing studies have used the external KAM. We have now clarified this in the discussion.

Nonetheless, we have contrasted our findings with the relevant existing literature (discussion - lines 379 – 399) and suggested potential hypothesis as to why our findings with MTCF estimates conflict with the past KAM literature. We also extracted KAM estimates from our modelling to demonstrate comparable findings to the existing literature (Table 2).

Another reason for the scarcity of relevant literature is due to the heterogeneity in shoe types evaluated. For example. We considered comparing our results to Kutzner et al. (2013 *J Biomech* 10.1016/j.jbiomech.2012.11.020*),* who evaluated in-vivo loads during walking in various running shoes with instrumented knee implants. However, there was no true flat flexible shoe in this study, which makes it very difficult to compare our findings.

As per our discussion (lines 427-433), most research investigating the influence of stable supportive and flat flexible shoes is limited to running in healthy, young people. This research cannot be used to compare findings because (i) it is a different task; (ii) footstrike position varies in running, while heel-strike is constant during walking; (iii) walking and running speeds differ substantially which will affect in vivo loads, and iv) the population in these studies do not have knee OA and running is not a functional task of relevance to most people with painful malaligned osteoarthritic knees.

Nonetheless, we have extended our discussion using additional literature where possible, with a focus on mechanisms that may explain our findings.

**Amendment**:

Discussion – Lines 377-379: “As this is the first study to evaluate the effects of stable supportive and flat flexible shoes on MTCF during walking using EMG-informed neuromusculoskeletal modelling, direct comparison with existing literature is precluded.”

Discussion – Lines 403-409: “Based on our findings, we speculate that at peak MTCF there was a concomitant increase in muscle activity across the knee, which nullified the decrease in external loads in flat flexible shoes compared to stable supportive shoes. Limited evidence has demonstrated higher medial gastrocnemius and gluteus maximus activation during the stance phase of running in acute bouts of barefoot compared to shod running (45). Indeed, it is plausible that flat flexible shoes may increase proximal activation during walking in knee OA.”

Discussion – Lines 411-414: “Furthermore, variations in tibia, rearfoot and forefoot motion have been shown to influence external knee loads (47). However, the influence of flat flexible and stable supportive shoes on these parameters were not evaluated in this study.”

Discussion – Lines 448-457: “Interestingly, “athletic”, “sturdy/supportive” and “cushioned” shoes are most frequently worn by people with knee OA, with both clinicians and patients believing that they are the most suitable (53). However, these characteristics are not mutually exclusive to stable supportive shoes. A rigorous RCT comparing stable supportive to flat flexible shoes reported 88% of participants wore shoes with mixed properties before enrolment and had comparable treatment expectations at baseline (11). Based on these findings, it is unclear whether the lower MTCF with stable supportive shoes observed in this study stems from habitual walking in this shoe type. Indeed, study of longitudinal responses of MTCF to footwear needs to be undertaken to understand any muscular, gait or habitual adaptations.”

**Reviewer #2:** I suggest deleting the first two sentences, adding a review of the main objective of the study and the main findings at the beginning of the first paragraph.

**Response:** We have implemented this change as suggested.

**Amendment**:

Discussion – Line 365-368: **“**The primary objective of this study was to use EMG-informed neuromusculoskeletal modelling to compare the effects of flat flexible to stable supportive walking shoes on MTCF waveforms and discrete measures of MTCF (peak, impulse and loading rate) in people with medial knee OA and varus malalignment.”

**Reviewer #2:** Lines 350-352 “Inspection of Figure 3 waveforms suggest that at peak MTCF the muscle component to MTCF was higher, and the external component to MTCF lower, in flat flexible shoes compared to stable supportive shoes.”. It is an important result that goes in the opposite direction to what is presented at the conclusion of the study, as I understand that this relationship brings an important advantage to the flat flexible shoes. A more consistent discussion of this relationship is needed.

**Response:**

A larger muscle contribution to MTCF (i.e., larger moments of muscles acting on the medial tibiofemoral compartment) as demonstrated in Fig 3 increases medial contact force (Winby et al., 2009 *J Biomech* 10.1016/j.jbiomech.2009.06.019). This is undesirable in people with knee OA, particularly if there is not an equal, or larger decrease in external loads as the net MTCF will have increased or remained the same.

We have now reworded this paragraph for clarity.

**Amendment**:

Discussion – line 401-409: “Inspection of Fig 3 waveforms suggest that, at peak MTCF, the muscle component to MTCF was higher and the external component to MTCF lower in flat flexible shoes compared to stable supportive shoes. Based on our findings, we speculate that at peak MTCF there was a concomitant increase in muscle activity across the knee, which nullified the decrease in external loads in flat flexible shoes compared to stable supportive shoes. Limited evidence has demonstrated higher medial gastrocnemius and gluteus maximus activation during the stance phase of running in acute bouts of barefoot running compared to shod running (45). Indeed, it is plausible that flat flexible shoes may increase proximal activation during walking in knee OA.”

**Reviewer #2:** A fundamental discussion point can be developed from a practical and evolutionary view. It is important to consider that, normally, since we started walking, we use shoes that can be considered as stable supportive shoes and the use of flat flexible shoes may not provide the most natural condition for the development of walking. I strongly suggest reading the article. Lieberman DE, Venkadesan M, Werbel WA, Daoud AI, D'Andrea S, Davis IS, Mang'eni RO, Pitsiladis Y. Foot strike patterns and collision forces in habitually barefoot versus shod runners. Nature. 2010 Jan 28;463(7280):531-5. doi: 10.1038/nature08723. PMID: 20111000.

A link to this discussion can be made from the idea presented in lines 388-389 "Study of longitudinal responses of MTCF to footwear needs to be developed to understand any muscle or gait adaptations.", considering the "biomechanical mechanisms" under a perspective evolution of the type of shoes used.

**Response:**

We thank the reviewer for this point. Unfortunately, the goal of our paper was not to explore the evolutionary perspective of foot motion, footwear, or development, particularly given the vastly different terrains, surfaces and purposes of locomotion in contemporary society. Indeed, the cross-sectional design of our study does not allow any speculation regarding these issues.

Given the paper highlighted by the Reviewer discusses differences in landing techniques dependent on shoe type in young, healthy runners, it is not relevant to include in the discussion of our study’s findings where participants are aged over 50 and have symptomatic knee OA and varus malalignment. Instead, we have now referenced studies by Paterson et al. 2014 (*J Foot Ankle Res* 10.1186/s13047-014-0043-8*)* and Paterson et al. 2021 (*Ann Intern Med* 10.7326/M20-6321) where habitual footwear use and beliefs are considered in people with knee OA.

We have, however, included the Lieberman paper as a reference in line 430, where we discuss the challenges of extrapolating biomechanical studies that examine running to those that examine walking.

**Amendment**:

Discussion – Line 427-433: “Unfortunately, other research comparing the effects of stable supportive and flat flexible shoe styles on loading rates is limited to running in young, healthy populations (22), which makes direct comparisons with our findings in walking difficult given that loading rates during running are influenced by both foot strike pattern (49-51) and footwear style (20, 50). Furthermore, it is challenging to compare a clinical population aged ≥50 years with symptomatic medial knee OA and varus malalignment to a young population with asymptomatic knees due to disease-specific biomechanical strategies (20, 25).”

Discussion – Line 448-457: “Interestingly, “athletic”, “sturdy/supportive” and “cushioned” shoes are most frequently worn by people with knee OA, with both clinicians and patients believing that they are the most suitable (53). However, these characteristics are not mutually exclusive to stable supportive shoes. A rigorous RCT comparing stable supportive to flat flexible shoes reported 88% of participants wore shoes with mixed properties before enrolment and had comparable treatment expectations at baseline (11). Based on these findings, it is unclear whether the lower MTCF with stable supportive shoes observed in this study stems from habitual walking in this shoe type. Indeed, study of longitudinal responses of MTCF to footwear needs to be undertaken to understand any muscular, gait or habitual adaptations.”

**Reviewer #2:** Review the list of references, especially regarding the writing pattern for citing each journal, for example: “Gait Posture”; “Gait & Posture”. As well as the abbreviations of each journal.

**Response:** Thank you. We have now reviewed and edited the reference list to be consistent with PLOS One referencing guidelines.
